# Transfer Parameter Analysis of Chloride Ingress into Concrete Based on Long-Term Exposure Tests in China’s Coastal Region

**DOI:** 10.3390/ma15238517

**Published:** 2022-11-29

**Authors:** Zhihong Fan, Dagen Su, Zhijie Zhang, Mingfeng Zhong, Xinxing Zhang, Jianbo Xiong, Pengping Li

**Affiliations:** 1 School of Materials Science and Engineering, South China University of Technology, Guangzhou 510641, China; 2 Key Laboratory of Harbor & Marine Structure Durability Technology, Ministry of Communications, Guangzhou 510641, China

**Keywords:** concrete, durability, chloride diffusion coefficient, exposure test, RCM

## Abstract

Chloride penetration resistance is one of the most important performance measures for the evaluation of the durability of concrete under a chloride environment. Due to differences in theory and experimental conditions, the durability index (chloride diffusion coefficient) obtained from laboratory accelerated migration tests cannot reflect the real process of chloride ingress into concrete in the natural environment. The difference in test methods must be considered and the transfer parameter $k_{t}$ should be introduced into the service life prediction model when the test results of accelerated methods are used. According to the test data of coastal exposure in South China, the attenuation rule of the chloride diffusion coefficient of different cement-based materials changed with time and was analyzed in this paper. Based on the diffusion coefficient–time curve, the theoretical natural diffusion coefficients of 28 d and 56 d were deduced, which were compared with the chloride diffusion coefficients obtained from the non-steady-state rapid migration method (RCM) at the same age. Therefore, the transfer parameter $k_{t}$ that expounds the relationship between concrete resistance to chloride permeability under a non-stationary electrical accelerated state and natural diffusion in the marine environment can be calculated; thus, the RCM testing index can be used to evaluate the long-term performance of the concrete structure in the marine environment. The results show that the value of $k_{t}$ was related to environmental conditions, test methods, and binder systems.

## 1. Introduction

Chlorine ion transmission in concrete and induced steel corrosion are the main causes leading to the damage of coastal reinforced concrete structures. The durability and lifetime of the concrete structures in chloride environments depend on the anti-chloride ion permeability of concrete; therefore, the diffusion coefficient of chloride ions is one of the most important indicators in measuring the capability of concrete resisting chloride erosion. According to the test principles and test conditions, two types of diffusion for chloride in concrete can be categorized. One is the natural diffusion coefficient, which reflects the long-term transmission of chloride into concrete and can be directly used for durability design and service life prediction for marine concrete structures. Extensive research has been carried out on the natural diffusion coefficients [1,2,3,4,5]. In this research, concrete specimens were exposed at marine locations, and chloride concentration profiles in the natural environment were measured on specimens exposed for specific periods [6,7,8]. Through exposure, tests continued for a long time, and the influences of fly ash, silica fume, and the water binder ratio on chloride diffusion in concrete were analyzed, as well as factors such as temperature and salinity [9,10,11,12,13].

The second is the chloride diffusion coefficient obtained from the accelerated test, including the electrical non-steady-state migration coefficients, the apparent diffusion coefficient of the salt ponding test, and a variety of steady and non-steady-state chloride migration coefficients proposed by Wang and Xu [14]. Those diffusion coefficients are mainly used for evaluating the durability of concrete. Su and Niu [15] studied the relationship between salt ponding and accelerated chloride migration tests. The experimental data of Wang [16] showed that the value of a steady diffusion coefficient is lower by almost an order of magnitude than non-steady-state ones. Spiesz and Brouwers [17] studied the apparent and effective chloride migration coefficients. The diffusion coefficient was computed using the Nernst–Einstein relationship between electrical material properties and ionic diffusion by Petr Konečný [18].

In summary, existing comparative studies of diffusion coefficients focused on differences between a variety of coefficients obtained from laboratory tests with standard methods, and, generally, the steady-state diffusion coefficients were specified as the input parameter to the durability life prediction of concrete, lacking in consideration for the disparity between laboratory rapid test results and the long-term performance of concrete in the natural environment. Due to the difference in temperature and humidity, salinity, chloride ion adsorption, and the saturation state of concrete, the chloride diffusion (or migration) coefficients obtained by laboratory tests can hardly reflect the chloride ingress process of concrete in natural marine situations. Wang and Fu [7] deemed that the main reason for the difference between Dins (the instantaneous chloride diffusion coefficient determined by the chloride natural diffusion test) and DRCM (the chloride diffusion coefficient DRCM measured from the rapid chloride migration test) is that the process of chloride diffusion in concrete is influenced by convection and chloride binding during the chloride natural diffusion test, which can be ignored in the RCM test. In contrast, the chloride diffusion coefficients obtained from exposure tests in the real marine environment could be applied to describe the actual state of chloride diffusion in concrete structures, and those exposure coefficients can be used directly for the durability life calculation. Therefore, the accelerated diffusion coefficient cannot be used directly in the service life prediction for the reinforced concretes under natural chloride environments, unless the differences between the accelerated test conditions and the actual environmental conditions are considered and the conversion factors are recommended between the apparent diffusion coefficients and the natural diffusion coefficients for long-term performance.

In 2002, we started to place concrete test specimens at the Exposure Test Station in Zhanjiang in southern China. The natural chlorine diffusion coefficients at different exposure durations were measured, and the relationship between the natural chloride diffusion coefficient and the exposure duration in the actual marine environments was established. We also compared the natural chloride diffusion coefficients of specific exposure duration with the apparent diffusion coefficients, which were obtained by applying an external electric field to drive the chloride ions in the concretes of the same compositions. By analyzing the diffusion coefficients from different test methods, we obtained direct conversion factors to rapidly characterize the long-term performance of concrete durability by the accelerated electromigration method.

## 2. Materials and Experiments

### 2.1. Test Materials and Compositions

The tested binder materials include Portland cement (P·II.42.5), fly ash (FAII), and ground blast furnace slag (GGBS, S95). Due to the long interval between the two tests, two different binders were used. Table 1, Table 2 and Table 3 list the chemical compositions and physical properties of the concrete binders.

The concrete aggregates include crushed granite with 5~25 mm continuous gradation and river sand grains with a fineness modulus of 2.60. FDN-5 high-efficiency water-reducing agent, with its main components being the condensation polymer of β-naphthalene sulfonic acid and formaldehyde, is used to reduce water by > 20%.

Concrete specimens were cut into cubes of 100mm to investigate the chloride ingress of each mix proportion over a specified period. Concrete was designed using Portland cement, fly ash, and GGBS, mostly with water-to-binder ratios (W/B) of 0.35 (Table 4). Cylindrical concrete specimens measuring 100 mm in diameter and 200 mm in height with the same mixture were also prepared.

### 2.2. Non-Steady-State Rapid Chloride Migration Tests

**Figure 1:**
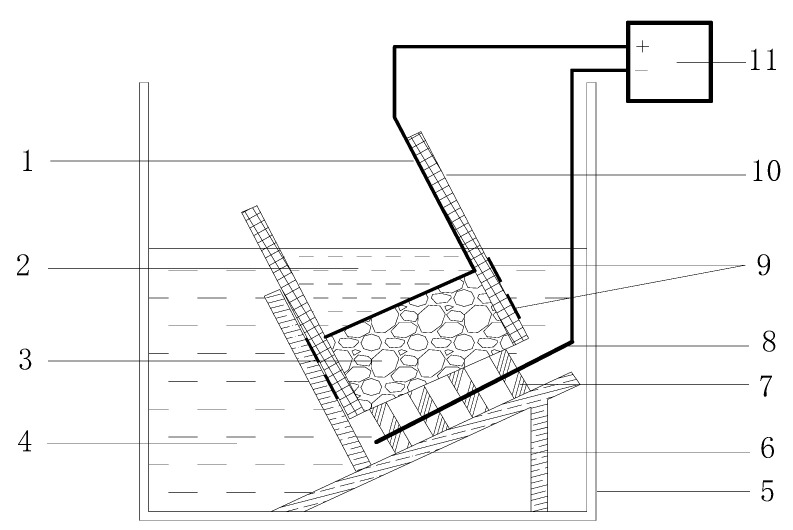
Test arrangement of the rapid chloride migration test (RCM). 1. Anode 2. Anolyte 3. Specimen 4. Catholyte 5. Plastice box 6. Plastic support 7. Cathod support 8. Cathod 9. stainless steel clamp 10. Rubber tube 11. Potential.

The specimens used were concrete rods with a diameter of 100 mm and a height of 50 mm. The concrete specimens were placed on the plastic support in the catholyte reservoir, which was filled with about 12 L of 10% NaCl solution. The sleeve was filled above the specimen with 300 mL anolyte solution (0.3 M NaOH). The test device is shown in Figure 1.

### 2.3. Exposure Test

**Figure 2:**
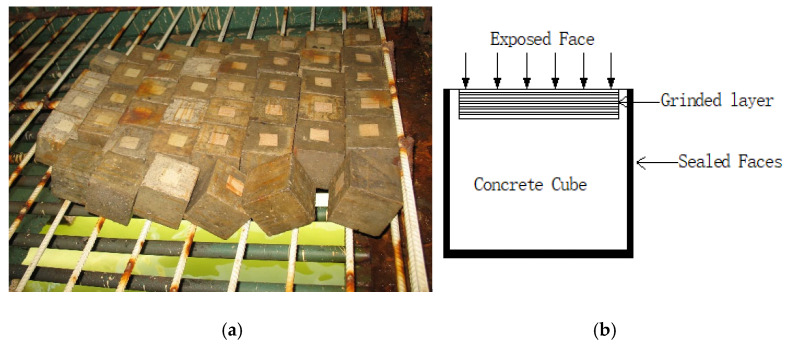
(**a**) Concrete specimens at the exposure test station and (**b**) Schematic diagram of concrete specimen testing.

Cubic concrete specimens (100 mm × 100 mm × 100 mm) were first removed from molds one day after being cast and were then cured in fresh water at 20 °C for 28 days. As shown in Figure 2, five surfaces of the cube were sealed up by epoxy resin, leaving only one surface exposed. The cubic specimens were transferred to the splash zone (with alternating wet and dry areas) of the marine exposure testing station in Zhanjiang City, South China. The annual average temperature at this site is 23.5 °C. Chemical analysis of the seawater found an average chloride content of 15,100 mg/L. Chloride tests were conducted to measure the chloride ingress at different depths (1~2 mm/layer) in each concrete specimen after the cubic concrete specimens were exposed to seawater for 90 days, 180 days, and 1, 2, 4, 7, and 10 years (1, 2, 4, 7, 10a). Concrete specimens were ground in the direction parallel to the exposed surface. The powder samples of each layer (1~2 mm/layer) were collected and digested by acid. The chloride content was then determined by titration for each layer at different depths. The apparent chloride diffusion coefficients of different ages were calculated by Fick’s second law.

### 2.4. Concrete Pore Structure Test (MIP)

The concrete was broken into particles smaller than 4.75 mm, and the particles with diameters between 2.36 mm and 4.75 mm were screened for the mercury injection test. The PASCAL140 and 240 automatic mercury injectors manufactured by Thermoelectric Finnegan Italy were used.

## 3. Transfer Parameter Analysis

### 3.1. Age Effect of Chloride Diffusion Coefficients

**Figure 3:**
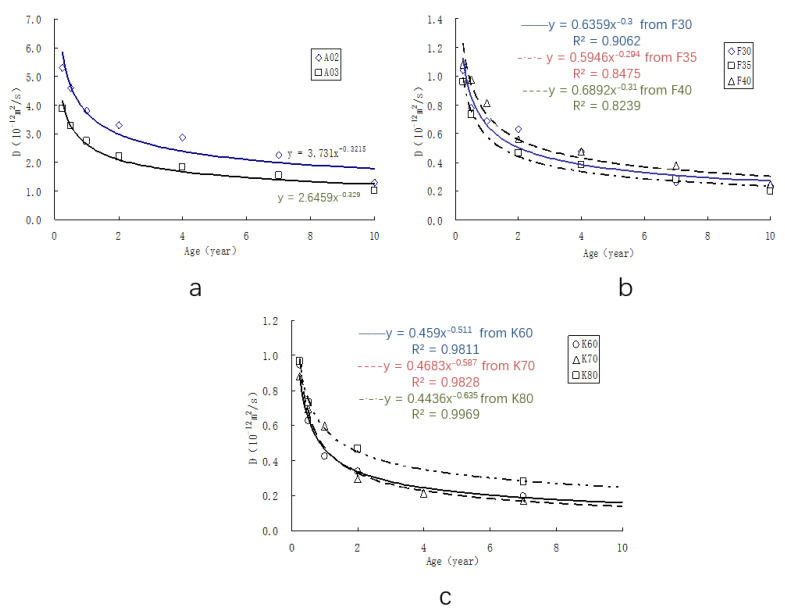
Chloride diffusion coefficient–time curves of concrete with different mineral admixtures based on exposure. (**a**) Portland cement concrete, (**b**) FA concrete, (**c**) GGBS concrete.

Due to the continued hydration of the binder, concrete permeability decreases over time. From the 10-year exposure test results in South China, the relationship between time and the chloride diffusion coefficients of concrete in the actual marine environment can be obtained, as shown in Figure 3.

Figure 3 shows the exponential attenuation of the chloride diffusion coefficient over time for concrete’s long-term performance. The type of activity admixture has the greatest impact on attenuation, followed by the water-to-binder ratio. The dosage of admixture influences the decay rate also.

Takewaka and Mastumoto [19] proposed the time dependence of the chloride diffusion coefficient of concrete. In the ACI software “Life365”, the following equation [20] was suggested to describe the relationship between time and chloride diffusion coefficient and to predict the service lifetime:

(1) $$D_{t}=D_{ref}\cdot {\left(\frac{t_{ref}}{t}\right)}^{m}$$

where *D_t_* is the chloride diffusion coefficient at time *t*, *D_ref_* is the chloride diffusion coefficient at time *t_ref_*, and *m* is the age factor of the chloride diffusion coefficient.

Figure 3 validates the description of Equation (1). Through fitting the regression curve in Figure 3, the age factor *m* of the diffusion coefficient can be derived for concrete with different cementitious materials, and the water-to-binder ratio is 0.35 in South China coastal areas of the marine environment (Table 5). Concrete with ground blast furnace slag has the maximum value of *m*, which means the diffusion coefficient decreases faster with time if using GGBS, and the concrete has an excellent long-term anti-chloride permeability performance.

The age factor *m* was expressed in different forms according to the study of Duracrete, Fib, and Life365 on the durability design model of concrete structures. Based on the concrete proportion, the value of *m* could be calculated for different models (Table 6). Comparing the data in Table 5 with those in Table 6, a similarity can be observed between the values of the age factor, but differences are also visible which may be caused by the constituents of the concrete materials, environment conditions, test methods, and analytical methods.

### 3.2. Diffusion and Migration Coefficients at Same Ages

According to the curvilinear relationship in Figure 3, Equation (1) can be used to derive the natural diffusion coefficient (*De*) of concrete at 28 days and 56 days, which corresponds to long-term resistance to chloride permeability for concrete in marine environments in South China.

With the development of testing technologies, the non-steady-state electro-migration method (RCM method) becomes the main method to measure the chloride ion resistance of the concrete in China. The Hong Kong–Zhuhai–Macau Bridge and many other large projects applied this method as the standard method for the quality control of concrete durability [26,27]. To correlate the durability index between the accelerated field test and long-term performance test, concrete specimens with the same mix and similar raw materials as the exposed specimens were molded again, and the chloride diffusion coefficient (*D_R_*) was measured through an RCM experiment after curing in standard conditions for 28 days and 56 days. All the diffusion coefficients obtained from exposure and RCM are shown in Table 7.

According to Table 7, chloride diffusion coefficients obtained from RCM testing methods, namely, under the condition of electric acceleration, are significantly greater than the natural diffusion coefficients for long-term exposure tests in South China.

The coefficients *De* and *D_R_* have a similar value for concrete without an active admixture at the same age, meaning that the results of the non-steady-state electric acceleration test based on the Nernst–Planck equation can reflect the short-term durability indicators and long-term performance when not considering the influence of an active admixture.

Due to the secondary hydration of the active admixture of fly ash and GGBS, the concrete structure tends to be denser over time, and the concrete durability improves. Therefore, the natural diffusion coefficient *De* is smaller than *D_R_*. Since the accelerated tests hardly reflect the secondary hydration effect on durability improvement, the diffusion coefficients *D_R_* of 56 days are closer to the natural diffusion coefficients compared to the 28-day test results.

### 3.3. Concrete Pore Structure at Different Ages

The pore structure characteristics of concrete are the most important factor affecting the chloride ion transport in concrete. Table 8 shows the pore structure of concrete at the ages of 28 days, 180 days, and 12 years. It can be seen that, with the extension of time, the pore diameter distribution of the concrete obviously moves to the direction of small size, and after using fly ash or GGBS, the pore diameter refinement of concrete is particularly obvious. According to the pore theory of concrete, different sizes of pores affect different properties, and the permeability of chloride ions mainly depend on pores with sizes ranging from 50nm to 10μm. As can be seen from Table 8, after the incorporation of FA or GGBS, the number of gel pores in concrete increases after 12 years, and the number of capillary pores decreases more obviously.

### 3.4. Relationship between RCM Diffusion and Long-Term Durability

The effective chloride diffusion coefficients used to predict the service life of reinforced and non-cracked concrete structures exposed to chloride is an indicator of the long-term durability of concrete. The steady diffusion coefficients after long-term decrescence can be calculated by using the following equation based on Equation (1):

(2) $$D_{app}(t)=D_{R}(t_{ref})\cdot k_{t}\cdot k_{e}\cdot {\left(\frac{t_{ref}}{t}\right)}^{m}$$

where *D_app_* (*t*) is the effectual diffusion coefficient at time *t* used for life prediction, *D_R_* (*t_ref_*) is the apparent diffusion coefficient at time *t_ref_* measured by the RCM test, *kt* is the transfer parameter, *ke* is the environmental variable, *t* is the time when the diffusion coefficient becomes stable (usually 20 years), and *m* is the age factor.

**Figure 4:**
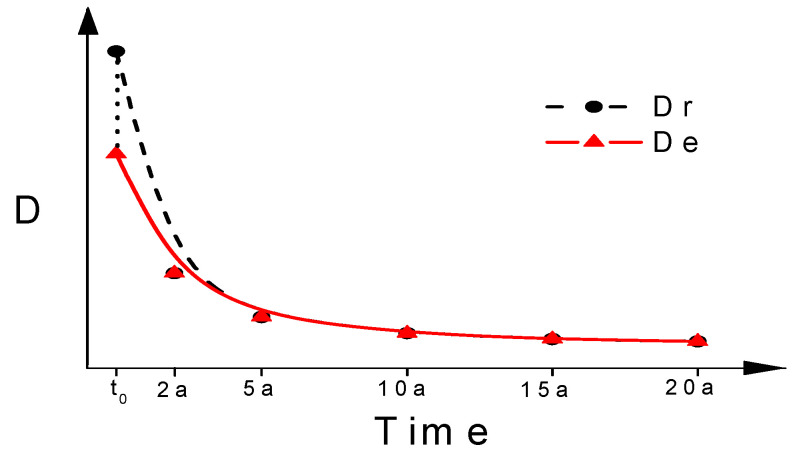
Relationship between diffusion coefficient of RCM and exposure test.

The age factor cannot be measured by the rapid RCM test method, because RCM tests at different ages represent only a certain portion of the total effect (an increase in chloride penetration resistance due to ongoing hydration of concrete), without considering the external influencing factors such as tide, temperature and so on [28]. In some durability life prediction models, the RCM diffusion coefficient at a specific age (*t*_0_) is plotted together with the exposure test results, and a regression line (dotted line in Figure 4) is established. In this case, the age factor *m* in Equation (2) is obtained through regression analysis, where the transfer parameter *kt* is 1. The disadvantage of this approach is the introduction of an accelerated index for the electric field to describe the long-term migration of chloride ions, resulting in a deviation from the attenuation theory. In this paper, natural diffusion coefficients at *t*_0_ are calculated according to exposure regression curves firstly (solid line in Figure 4), and then the RCM diffusion coefficients at *t*_0_ are converted to the natural diffusion coefficient through the transfer parameter *kt* to reflect the influence of the test methods (dotted line in Figure 4). That approach is logically clearer.

Therefore, the age factor *m* can be shown in Table 5 for concrete with a water-to-binder ratio of 0.35 in the environmental conditions of the South China coastal area. The value of transfer parameter *kt* can be obtained by a coefficients analysis between natural diffusion and rapid tests, according to Table 7.

**Figure 5:**
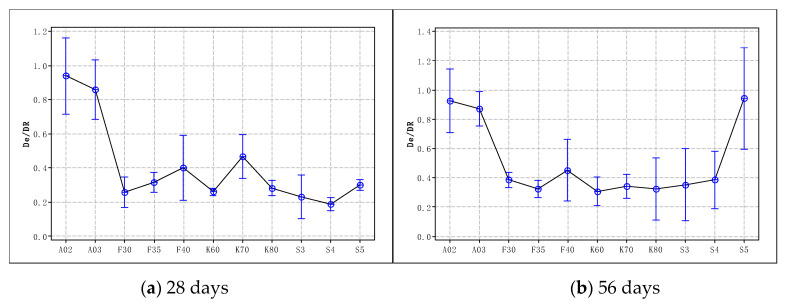
Ratios of diffusion coefficients of 28 and 56 days.

The relationships between *D_R_* and *De* at the age of 28 days and 56 days are shown in Figure 5. For Portland cement, *kt* is approximately 0.9. After using activated admixtures, the value is related to the age, type, and dosage of the admixture. For fly ash concrete, *kt* is in the range of 0.25 to 0.31 (28 d) or 0.32 to 0.43 (56 d). For GGBS concrete, *kt* is in the range of 0.26 to 0.48 (28 d) or 0.30 to 0.35 (56 d).

The chloride diffusion coefficient is concentration-dependent, while the RCM method uses a 10% chloride concentration solution, which is much higher than the chloride concentration in natural seawater (generally no more than 2%), resulting in a *D_R_* of the same age greater than the *De*. In addition, the RCM method is based on electric acceleration, and the selection of the initial current of the applied electric field is affected by the pore structure. Referring to Table 8, the average pore diameters of the FA concrete and GGBS concrete are smaller than that of concrete without an admixture, which means that the initial current is small, and the amount of chloride migrated under the electric field is not too big and does not lead to a small result. Finally, the RCM method has a short test period, and it is difficult to reflect the impact of chloride ion adsorption on the test results. The short-age diffusion coefficient *De* derived from the long-term natural diffusion coefficient fully reflects the adsorption effect of FA and GGBS on chloride ions. Therefore, the *kt* of FA concrete or GGBS concrete is much smaller than that of Portland cement, and, according to the comparison of pore structure parameters and the analysis of the chloride ion adsorption effect, the values of the FA concrete and GGBS concrete are very close.

The discussion above is based on the comparison of RCM results with the exposure diffusion coefficients in the South China coastal environment; therefore, the value of *kt* might be only adequate for coefficient conversion and durability design for similar environmental conditions in the South China coastal area. For other environmental conditions, such as cold areas, the transfer parameter *kt* should be reanalyzed by considering the influence of temperature differences and frozen thaw and be amended by the test results in areas exposed to cold.

## 4. Conclusions

(1) The age factor *m* of the chloride diffusion coefficient is calculated based on the 10 years of data from marine exposure tests in South China.
(2) With the extension of age, the pore diameter distribution of the concrete shifts to the direction of small size. Moreover, using FA or GGBS refines the pore diameter of concrete. At 12-years-old, the gel hole of concrete with FA or GGBS is more than 50% of the total pores.
(3) Chloride diffusion coefficients measured by the electric field accelerated method (RCM) are significantly greater than the natural diffusion coefficients at the same age, being calculated from the long-term exposure test results in South China. Since short-time accelerated test results do not reflect the effect of secondary hydration caused by active admixtures on concrete pore structure and chloride ion adsorption improvement, there is a difference between the two diffusion coefficients, which are more obvious for concrete using FA and GGBS.
(4) The durability rapid test results should be converted to an effective chloride diffusion coefficient in the service lifetime prediction of reinforced concrete structures. The transfer parameter *kt* is related to the engineering environment conditions, test method, test age, and binder system. When the concrete structures are situated in a high-temperature and high-humidity environment, which is similar to South China, the transfer parameter *kt* can take the values of 0.9 for Portland cement concrete, 0.25~0.31 (28 d), and 0.32~0.43 (56 d) for FA concrete, and 0.26~0.48 (28 d) and 0.30~0.35 (56 d) for GGBS concrete.

## Figures and Tables

**Table 1:** Chemical Composition and Physical Properties of Cement.

	Chemical Composition (%)								Flexural Strength (MPa)		Compressive Strength (MPa)	
	SiO_2_	Fe_2_O_3_	Al_2_O_3_	CaO	MgO	f-CaO	SO_3_	Loss on Ignition	3 d	7 d	3 d	28 d
A	21.3	3.42	5.01	65.49	2.42	1.01	1.04	2.2	6.9	9	25.1	58.5
B	20.9	4.6	6.02	59.07	1.46	0.78	2.98	2.4	5.7	9.1	24.4	59.1

**Table 2:** Chemical Composition and Physical Properties of FA.

	Chemical Composition (%)								Density (g/cm^3^)	Fineness (%)	Water Requirement Ratio	Strength Activity Index
	SiO_2_	Fe_2_O_3_	Al_2_O_3_	CaO	MgO	f-CaO	SO_3_	Loss on Ignition				
A	60.8	5.76	24.12	3.03	0.55	0.32	0.63	3.6	2.1	6.8 *	98	75
B	56.3	4.92	25.78	6.91	0.76	0.31	0.3	2.9	2.2	4.9 *	97	72

* 45 μm square-hole sieve.

**Table 3:** Chemical Composition and Physical Properties of GGBS.

	Chemical Composition (%)			Density (g/cm^3^)	Specific Surface (m^2^/kg)	Activity Index (%)	
	SO_3_	Cl	Loss on Ignition			7 d	28 d
A	0.25	0.02	0.41	2.9	467	87.7	96.5
B	0.19	0.06	0.49	2.8	410	88	98

**Table 4:** Mixture Proportions of Concrete.

Mix No.	W/B	Binder (kg/m^3^)	FA (%)	GGBS (%)	Slump (mm)	28 Days Strength (MPa)
A02	0.40	438	0	0	186	60.5
A03	0.35	460	0	0	183	66.2
F30	0.35	457	30	0	188	55.7
F35	0.35	454	35	0	198	50.7
F40	0.35	454	40	0	200	51.6
K60	0.35	434	0	60	200	55.5
K70	0.35	434	0	70	210	53.3
K80	0.35	436	0	80	205	42.6

**Table 5:** The Age Factor of Concrete with Various Admixtures.

Binder	Cement	Cement + FA	Cement + GGBS
m	0.32	0.38	0.50

**Table 6:** The Age Factor of Chloride Diffusion Coefficient.

	Binder	Cement	Cement + FA	Cement + GGBS
Model				
Duracrete [21,22]		0.37	0.93	0.60
Fib [23]		Beta (m ^a^ = 0.30,s ^b^ = 0.12)	Beta (m ^a^ = 0.60, s ^b^ = 0.15)	Beta (m ^a^ = 0.45, s ^b^ = 0.20)
Life365 [24,25]		0.2	0.44–0.52	0.54–0.66

^a^ m: mean value; ^b^ s: standard deviation.

**Table 7:** Diffusion Coefficients are Calculated from Different Methods.

Types of Binder	Concrete Age	28 Days (10^−12 ^m^2^/s)		56 Days (10^−12 ^m^2^/s)	
	Mix No.	*D*_R_	*D*_e_	*D*_R_	*D*_e_
Cement	A02	9.24	8.65	7.52	6.92
	A03	7.38	6.31	5.79	5.04
Cement + FA	F30	7.28	1.85	3.69	1.42
	F35	5.24	1.65	3.90	1.26
	F40	6.68	1.91	3.42	1.47
Cement + GGBS	K60	4.46	1.16	2.92	0.89
	K70	3.56	1.65	3.36	1.14
	K80	5.39	1.52	3.78	1.18

**Table 8:** Characteristic Parameters of Pore Structure of Concrete at Different Ages.

Number	Age	Mean Pore Size (nm)	Total Porosity (%)	Pore Size Distribution (%)				
				<10 nm	10~50 nm	50~100 nm	100~1000 nm	>1000 nm
A03	28d	47.0	12.7	8.6	25.4	14.5	16.6	28.0
A03	0.5a	21.9	13.1	15.1	33.5	5.9	11.9	33.7
A03	12a	24.3	6.0	15.4	42.5	6.0	9.0	27.1
F30	28d	16.6	13.0	23.2	29.5	9.4	8.0	29.9
F30	0.5a	16.7	11.3	25.1	32.3	5.3	10.4	26.8
F30	12a	8.9	6.3	60.8	10.3	2.6	7.7	8.5
K60	28d	27.8	17.8	16.0	20.1	11.8	16.1	36.0
K60	0.5a	10.4	11.1	42.5	15.7	2.7	8.7	30.4
K60	12a	6.8	7.9	58.4	18.0	4.8	8.7	10.1

## Data Availability

Not applicable.

## References

[1] Zainasallehen S., Duraman S.B. (2018). Properties of PFA Concrete at Different Curing Conditions. IOP Conf. Ser. Mater. Sci. Eng..

[2] Runci A., Provis J., Serdar M. (2022). Microstructure as a key parameter for understanding chloride ingress in alkali-activated mortars. Cem. Concr. Compos..

[3] Cheewaket T., Jaturapitakkul C., Chalee W. (2014). Concrete durability presented by acceptable chloride level and chloride diffusion coefficient in concrete: 10-year results in marine site. Mater. Struct..

[4] Wang Y., Liu C., Tan Y., Wang Y., Li Q. (2020). Chloride binding capacity of green concrete mixed with fly ash or coal gangue in the marine environment. Constr. Build. Mater..

[5] Alexander M., Beushausen H. (2019). Durability, service life prediction, and modelling for reinforced concrete structures–review and critique. Cem. Concr. Res..

[6] Tang H., Yang Y., Peng J., Liu P., Zhang J. (2022). Test and Mesoscopic Analysis of Chloride Ion Diffusion of High-Performance-Concrete with Fly Ash and Silica Fume. Coatings.

[7] Wang Y., Fu K. (2019). Comparisons of instantaneous chloride diffusion coefficients determined by RCM method and chloride natural diffusion test. Const. Build. Mater..

[8] Bao J., Wei J., Zhang P., Zhuang Z., Zhao T. (2022). Experimental and theoretical investigation of chloride ingress into concrete exposed to real marine environment. Cem. Concr. Compos..

[9] Thomas M., Bremner T. (2012). Performance of lightweight aggregate concrete containing slag after 25 years in a harsh marine environment. Cem. Concr. Res..

[10] Moffatt E.G., Thomas M., Fahim A. (2017). Performance of high-volume fly ash concrete in marine environment. Cem. Concr. Res..

[11] Zhang J., Zhao J., Zhang Y., Gao Y., Zheng Y. (2018). Instantaneous chloride diffusion coefficient and its time dependency of concrete exposed to a marine tidal environment. Constr. Build. Mater..

[12] Wang Y., Wu L., Wang Y., Li Q., Xiao Z. (2018). Prediction model of long-term chloride diffusion into plain concrete considering the effect of the heterogeneity of materials exposed to marine tidal zone. Constr. Build. Mater..

[13] Wang J., Ng P., Su H., Du J. (2020). Influence of the coupled time and concrete stress effects on instantaneous chloride diffusion coefficient. Const. Build. Mater..

[14] Xu S., Li Q., Wu Y., Dong L., Lyu Y., Reinhardt H.W., Leung C.K.Y., Ruiz G., Kumar S., Hu S. (2021). Results of round-robin testing for determining the double-K fracture parameters for crack propagation in concrete: Technicalreport of the RILEM TC265-TDK. Mater. Struct..

[15] Su L., Niu D., Huang D., Luo Y., Qiao H., Zhang Y. (2022). Chloride diffusion behavior and microstructure of basalt-polypropylene hybrid fiber reinforced concrete in salt spray environment. Constr. Build. Mater..

[16] Wang R., He F., Chen C., Dai L. (2021). Coupling effect of the connected pores and pore solution on chloride ion migration in cement-based materials. Constr. Build. Mater..

[17] Spiesz P., Brouwers H. (2013). The apparent and effective chloride migration coefficients obtained in migration tests. Cem. Concr. Res..

[18] Konečný P., Lehner P., Ponikiewski T., Miera P. (2017). Comparison of Chloride Diffusion Coefficient Evaluation Based on Electrochemical Methods. Procedia Eng..

[19] Takewaka K., Mastumoto S. (1988). Quality and cover thickness of concrete based on the estimation of chloride penetration in marine environments. Spec. Publ..

[20] Thomas M.D.A., Bentz E.C. (2001). Life-365: Computer program for predicting the service life and life-cycle costs of reinforced concrete exposed to chlorides. Am. Concr. Inst. Comm..

[21] Wu L., Li W., Yu X. (2017). Time-dependent chloride penetration in concrete in marine environments. Constr. Build. Mater..

[22] Ding Y., Qin X., Zhou Y., Liu J., Shi L., Mu S. (2020). Durability life prediction of reinforced concrete structures under chloride ion erosion--comparison and analysis of classical models. Concrete.

[23] (2020). Structural Concrete; New Structural Concrete Findings from University of Natural Resources and Applied Life Science Reported (Fib Models for Modeling of Chloride Ion Ingress and Concrete Carbonation: Levels of Assessment of Input Parameters). J. Technol..

[24] Zhong W., Lu Z., Su Y. (2014). Application of Life-365 in the durability design of concrete for seaports. Water Transp. Eng..

[25] Han Y., Xie D., Zhang W., Wu W., Ma L. (2022). Durability design of concrete materials in chloride environment based on software Life-365. Ocean Lake Bull..

[26] Zhu Y., Lin M., Meng F., Liu X., Lin W. (2019). The Hong Kong–Zhuhai–Macao Bridge. Engineering.

[27] Wang S., Li K., Fan Z., Su Q., Xiong J. (2015). Countermeasures for 120a service life durability of main concrete structure of Hong Kong-Zhuhai-Macau Bridge. Water Transp. Eng..

[28] (2006). Model Code for Service Life Design.

